# The Effect of Cyanine Dye NK-4 on Photoreceptor Degeneration in a Rat Model of Early-Stage Retinitis Pigmentosa

**DOI:** 10.3390/ph14070694

**Published:** 2021-07-19

**Authors:** Shihui Liu, Toshihiko Matsuo, Mary Miyaji, Osamu Hosoya

**Affiliations:** 1 Department of Ophthalmology, Okayama University Graduate School of Interdisciplinary Science and Engineering in Health Systems, Okayama City 700-8558, Japan; shihuiliu@okayama-u.ac.jp; 2 Department of Medical Neurobiology, Okayama University Graduate School of Medicine, Dentistry and Pharmaceutical Sciences, Okayama City 700-8558, Japan; mmiyaji@okayama-u.ac.jp (M.M.); hosoya@okayama-u.ac.jp (O.H.)

**Keywords:** NK-4, retina, apoptosis, RNA-seq, photoreceptor, retinitis pigmentosa, antioxidant, metabolism

## Abstract

The present study aimed to evaluate the effects of NK-4 on the apoptosis of photoreceptors in a rat model of retinitis pigmentosa and explore the mechanism underlying anti-apoptosis activity. The Royal College of Surgeons (RCS) rats received an intravitreous injection of NK-4 solution in the left eye and vehicle control in the right eye. Apoptosis was detected by TUNEL method in frozen sections of the eyes. The retinal tissues of the rats were dissected for RNA-seq analysis. Functional and pathway enrichment analyses of differentially expressed genes (DEGs) were performed by using Metascape and DAVID software. The expression levels of DEGs were confirmed by real-time quantitative PCR (RT-qPCR). The number of apoptotic cells decreased in the outer nuclear layer (ONL) and the thickness of the ONL was significantly thicker in the retina of NK-4-injected eyes, compared with control eyes. Five DEGs were identified by RNA-seq analysis, and *Hmox1, Mt1, Atf5, Slc7a11,* and *Bdh2* were confirmed to be up-regulated by RT-qPCR. Functional and pathway enrichment analysis of the up-regulated genes showed that anti-apoptosis effects of NK-4 in the retina of RCS rats may be related to the pathways of metal ion homeostasis, negative regulation of neuron death, response to toxic substance, and pigment metabolic process. We found a potential mechanism of NK-4, providing a new viewpoint for the development of more therapeutic uses of NK-4 in the future.

## 1. Introduction

Retinitis pigmentosa (RP) is a group of inherited eye diseases due to the damage of retinal photoreceptor cells that convert light signals to electric signals, and mutations in more than 50 genes may contribute to the progression of RP [1]. Patients with RP experience the constriction of visual fields caused by rod dysfunction and finally lose the central vision around the macula [2]. The first gene therapy for *RPE65*-related inherited retinal diseases was successfully approved. There are many other treatments that are trying to rescue retina degeneration and improve visual function, such as artificial retina [3,4,5], retinal regeneration [6,7], antioxidants [8,9], and gene therapy [10].

We used the RCS rat as an animal model of RP because it shows progressive photoreceptor degeneration as the consequence of a primary retinal pigment epithelium (RPE) gene mutation. In the RCS rat, a 409 bp deletion in the receptor tyrosine kinase *Mertk* mutation results in defective phagocytosis of photoreceptor outer segments by the RPE, and the accumulation of debris leads to hypoxia [11,12]. Debris from apoptotic and dead cells, reactive oxygen species (ROS) and damaged lipopolysaccharides can trigger the loss of photoreceptor cells and retinal degeneration [13]. It was identified that RP patients have mutations in *Mertk* [14,15], and the photoreceptor cells have an inherent function. When the phagocytic function of RPE is recovered, they will continue to function [11,12]. This form of RP becomes an attractive target for the development of drug therapy protocols, and it has been demonstrated that interference of the process of apoptosis may slow down retinal degeneration [16].

**Figure 1:**
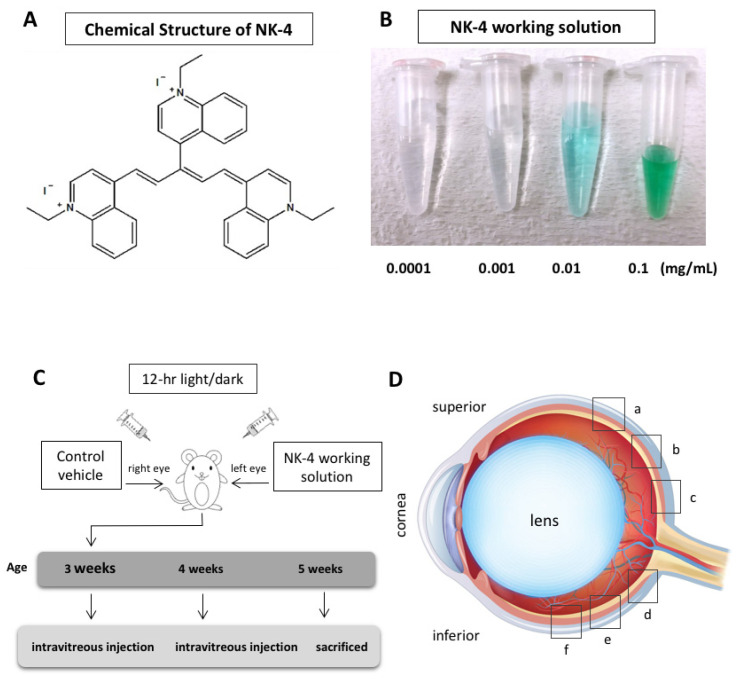
NK-4 and experimental design. (**A**) Chemical structure of NK-4. (**B**) A series of NK-4 working solutions with different concentrations (0.0001 to 0.1 mg/mL). (**C**) Experimental schedule. (**D**) Six retinal sites (a, b, c, d, e, and f) for TUNEL staining. The “a”, “b”, and “c” began at the straight distance of 586, 373, and 160 μm, respectively, superior from the optic nerve head, while “f”, “e”, and “d” began at the straight distance of 586, 373, and 160 μm, respectively, inferior from the optic nerve head.

NK-4 (IUPAC name: 1-ethyl-4-[(1Z,3E,5E)-1-(1-ethylquinolin-1-ium-4-yl)-5- (1-ethylquinolin-4-ylidene)penta-1,3-dien-3-yl]quinolin-1-ium;iodide) (Figure 1A) is a divalent cationic pentamethine trinuclear cyanine dye that includes three quinolinium rings, N-ethyl side chains, and two iodine anions. NK-4 is the common name of cryptocyanine O.A.1 (PubChem CID: 5489539) [17]. It has been studied for over 150 years in Japan and has been of research interest in biology and medicine [17,18,19,20,21,22,23]. NK-4 exhibits a variety of biological activities, such as preventing IL-4-driven polarization to alternatively activated macrophages and inhibiting cancer cell proliferation, allergy, and inflammation, and thus has been used to treat cancer and virus infection [17,18,19,20,21,22,23]. In addition, NK-4 acts as a significantly more potent radical scavenger than edaravone and ascorbate, especially for hydroxyl radicals, peroxy radicals, and superoxides [24]. NK-4 also exhibits potent neurotrophic effects via the activation of survival signal pathways [25,26,27], indicating its protective effects on nerve cells. However, it is still unclear whether NK-4 has neuroprotective effects on retinal cells.

Therefore, in this study, we aimed to evaluate the treatment effects of NK-4 on the RCS rat, a model of RP. In addition, we aimed to explore the mechanisms underlying the anti-apoptosis activity of NK-4 by RNA-seq and bioinformatics analyses.

## 2. Results

### 2.1. TUNEL Staining and ONL Thickness 

**Figure 2:**
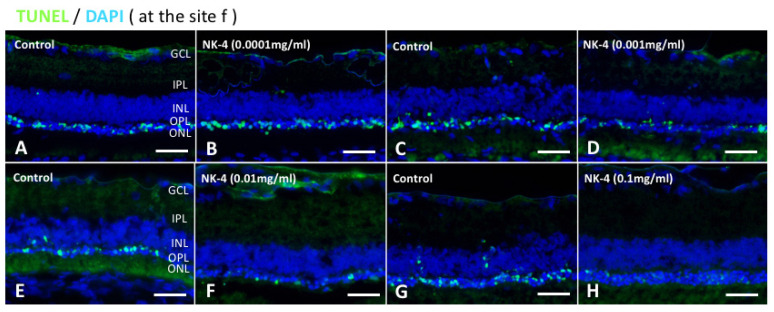
TUNEL staining of retinal sections at the site of “f”. TUNEL assay indicated that TUNEL-positive signals (green) were mainly detected in the ONL and were likely to be fewer in retinas treated with NK-4 (0.0001 mg/mL, 0.001 mg/mL, 0.01 mg/mL, and 0.1 mg/mL) than in those with control vehicle. Cell nuclei were counterstained with DAPI (blue). Pair groups: (**A**) vs. (**B**), (**C**) vs. (**D**), (**E**) vs. (**F**), (**G**) vs. (**H**). ONL, outer nuclear layer; INL, inner nuclear layer; OPL, outer plexiform layer. Scale bar = 25 μm.

**Figure 3:**
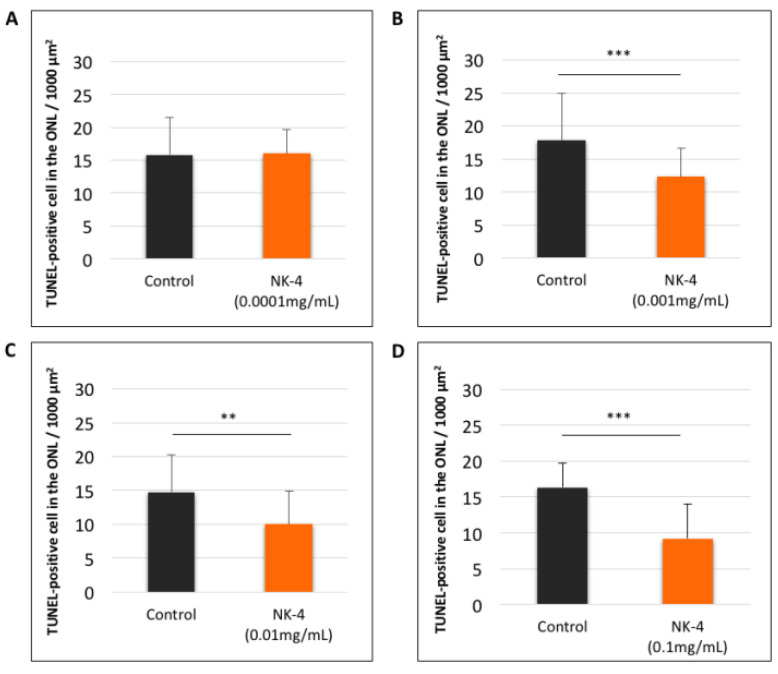
Quantitative analysis of TUNEL-positive cells (mean values of a, b, c, d, e, and f sites) in retinal sections. (**A**): NK-4 (0.0001 mg/mL)-injected retinas vs. control groups; (**B**): NK-4 (0.001 mg/mL)-injected retinas vs. control groups; (**C**): NK-4 (0.01 mg/mL)-injected retinas vs. control groups; (**D**): NK-4 (0.1 mg/mL)-injected retinas vs. control groups. The number of TUNEL-positive cells was significantly decreased in the ONL of the eyes injected with higher concentrations of NK-4, ranging from 0.001 to 0.1 mg/mL, compared with control vehicle-injected eyes (*p* < 0.0001, *p* = 0.001, *p* < 0.0001, respectively, one-factor ANOVA, post hoc test). **: p < 0.01, ***: p < 0.001.

We investigated the numbers of TUNEL-positive cells at different concentrations in NK-4 retinas, compared with control groups. The study areas of TUNEL staining were located in the sites a, b, c, d, e, and f (Figure 1D). In control vehicle-injected eyes, we found strong TUNEL-positive staining in the ONL where the nuclei of photoreceptor cells were located. In contrast, TUNEL-positive cells were fewer in the retinal sections from NK-4-injected eyes ( Figure 2; Figure 3). Figure 2 (site f) shows representative images of TUNEL staining, and other images (site a–e) are presented in Supplementary Figure S1 online. The numbers of TUNEL-positive cells (mean values of a, b, c, d, e, and f sites) were as follows: 0.0001 mg/mL NK-4: 16.04 ± 3.64, control: 15.85 ± 5.61; 0.001 mg/mL NK-4: 12.32 ± 4.35, control: 17.79 ± 7.11; 0.01 mg/mL NK-4: 10.05 ± 4.83, control: 14.72 ± 5.49; 0.1 mg/mL NK-4: 9.17 ± 4.79, control: 16.35 ± 3.39. The number of TUNEL-positive cells was significantly decreased in the ONL of the eyes injected with higher concentrations of NK-4, ranging from 0.001 to 0.1 mg/mL, compared with control vehicle-injected eyes (*p* < 0.0001, *p* = 0.001, *p* < 0.0001, respectively, one-factor ANOVA, post hoc test) (Figure 3).

**Figure 4:**
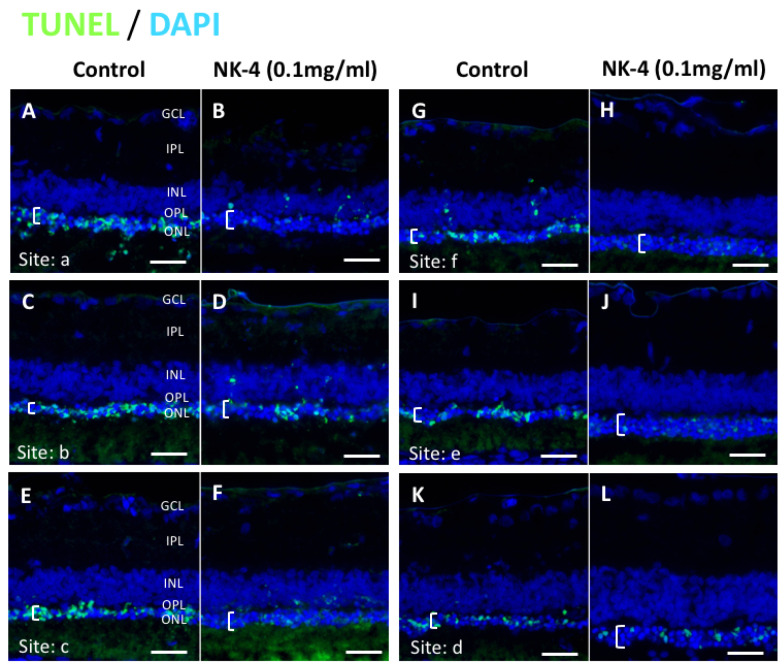
TUNEL staining of retinal sections at the concentration of 0.1 mg/mL NK-4 (this figure shows the condition of the entire retina in one RCS rat). The thickness of the ONL (white brackets) was thicker in the retina of NK-4-injected eyes, compared with control eyes. Cell nuclei were counterstained with DAPI (blue). Pair groups: (**A**) vs. (**B**), (**C**) vs. (**D**), (**E**) vs. (**F**), (**G**) vs. (**H**), (**I**) vs. (**J**), (**K**) vs. (**L**). ONL, outer nuclear layer; INL, inner nuclear layer; OPL, outer plexiform layer. White brackets represent the ONL. Scale bar = 20 μm.

**Figure 5:**
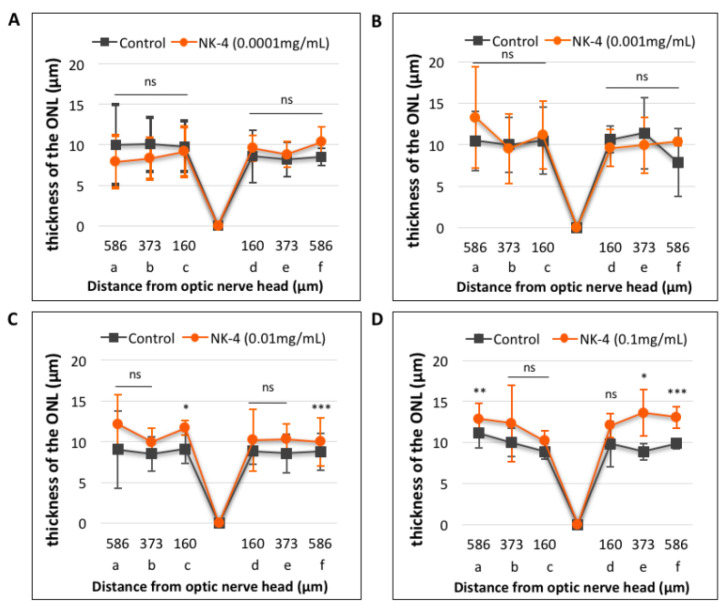
Quantitative analysis of the thickness of the ONL in retinal sections. (**A**): NK-4 (0.0001 mg/mL)-injected retinas vs. control groups; (**B**): NK-4 (0.001 mg/mL)-injected retinas vs. control groups; (**C**): NK-4 (0.01 mg/mL)-injected retinas vs. control groups; (**D**): NK-4 (0.1 mg/mL)-injected retinas vs. control groups. The thickness in the ONL: NK-4 (0.1 mg/mL) vs. control vehicle-injected eyes (site a: *p* = 0.00753, site e: *p*  =  0.01125, site f: *p* = 0.00067, Student’s *t*-test). The thickness in the ONL: NK-4 (0.01 mg/mL) vs. control vehicle-injected eyes (site c: *p* = 0.00344, site f: *p* = 0.00055, Student’s *t*-test). *: *p* < 0.05, **: *p* < 0.01, ***: *p* < 0.001.

Next, the changes in ONL thickness in the retina were investigated. Figure 4 shows representative images of NK-4 (0.1 mg/mL)-treated retinas. The thickness in the ONL was significantly different in eyes injected with NK-4 (0.1 mg/mL) compared with control vehicle-injected eyes (site a*: p*  = 0.00753, site e*: p*  =  0.01125, site f*:* *p* = 0.00067, Student’s t-test) (Figure 5). As shown in Figure 5D, *p* values at three locations (a, e, f) were significant; thus, NK-4 (0.1 mg/mL)-treated retinas and control groups were selected as representative. The thickness in the ONL was also significantly different in eyes injected with NK-4 (0.01 mg/mL) compared with control vehicle-injected eyes (site c*: p* = 0.00344, site f*: p* = 0.00055, Student’s t-test) (Figure 5).

### 2.2. Screening of Differentially Expressed Genes in The Eyes Injected with NK-4 

To reveal the mechanism by which NK-4 attenuates apoptosis in the retina, we examined gene expression changes in the retina samples. We isolated total RNA from retina samples and performed RNA-seq analysis. The total number of reads per sample ranged from 46.2 million to 68.6 million. We only focused on the genes with mean FPKM > 0.1 in each group to avoid genes with low expression, and we screened out a total of 13,132 expressed genes (see Supplementary Table S1 online). Genes with expression change ≥ 1.4-fold and assembled data of four sets with a *p* value < 0.05 were selected for follow-up studies, and 15 genes (*Atf5, Trib3, Nexn, Mt1, Casp4, Hmox1, Bfsp2, Gcg, Ppic, Cdh3, Slc22a8, Ppfibp2, Slc7a11, Bdh2, C1qtnf5*) were chosen for next analysis. Then, we checked the relevant literature to find the following five up-regulated genes as differentially expressed transcripts in the eyes treated with NK-4 that are related to anti-apoptosis: heme oxygenase 1 (*Hmox1)* [28], metallothionein-1 *(Mt1)* [29,30], *solute* carrier family 7 member 11 *(Slc7a11)* [31], 3-hydroxybutyrate dehydrogenase 2 *(Bdh2)* [32,33], and activating transcription factor 5 *(Atf5)* [34] (Table 1).

### 2.3. Bioinformatics Analysis of Differentially Expressed Genes in the Eyes Injected with NK-4 

**Figure 6:**
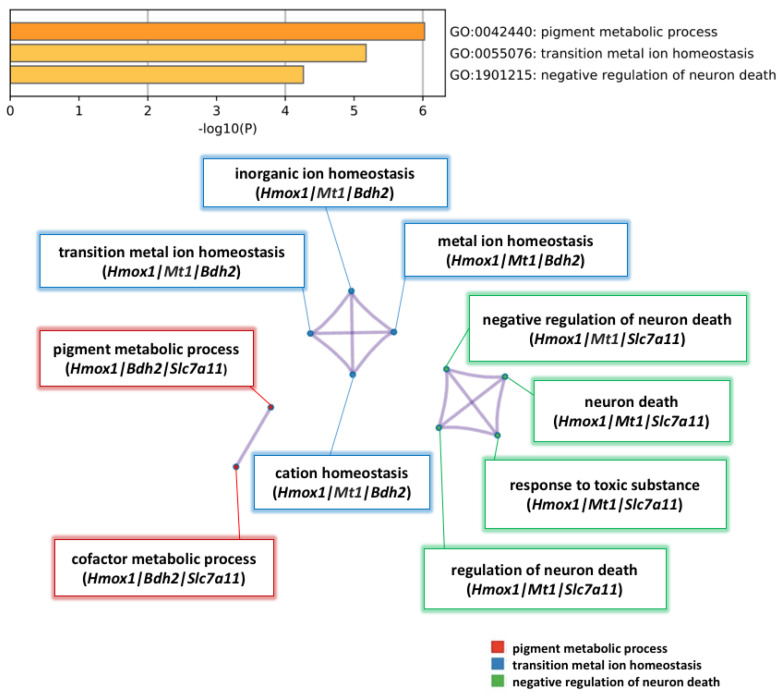
Pathway enrichment analysis of 5 significant genes up-regulated by NK-4. Heatmap of pathway enrichment analysis in the up-regulated genes. The three enriched terms in the category of biological process (BP) were pigment metabolic process, transition metal ion homeostasis, and negative regulation of neuron death. Nodes are clustered into subnetworks, and the included genes are shown under the respective pathways.

To analyze the pathways that contain the five selected genes (*Hmox1, Mt1, Slc7a11, Bdh2,* and *Atf5*), the DEG dataset was uploaded to Metascape for analysis. (See Supplementary Tables S2 and S3 online ). Significant pathways were associated with pigment metabolic process, transition metal ion homeostasis, and negative regulation of neuron death (Figure 6).

**Figure 7:**
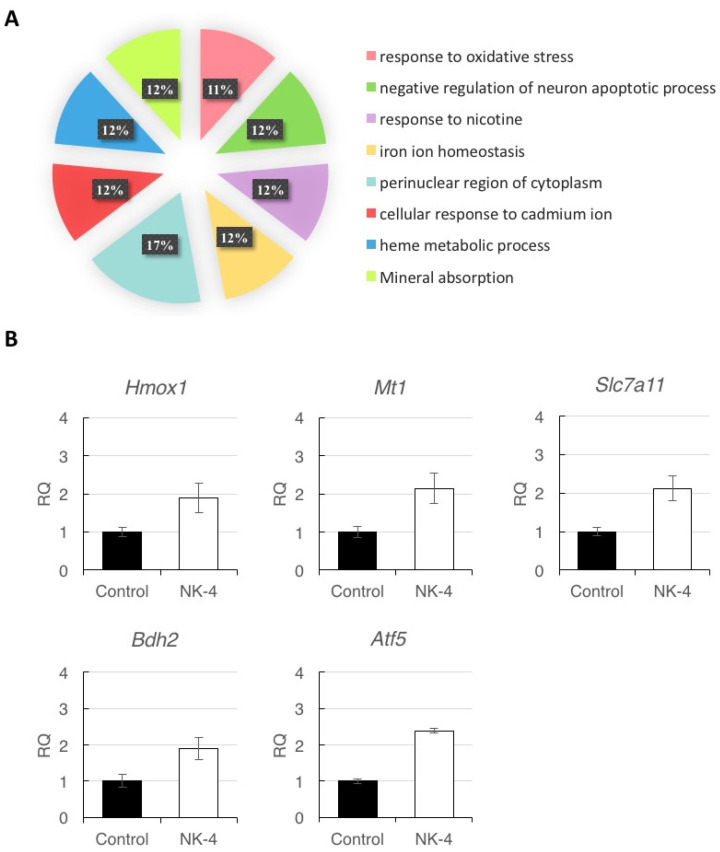
(**A**) Analysis of affected functional processes of DEGs. DEGs were submitted for DAVID analysis, and affected functional processes are shown. Significance was accepted at a Benjamini–Hochberg *p* value of less than 0.01. (**B**) Quantitative reverse-transcription PCR analysis of *Hmox1*, *Mt1*, *Slc7a11*, *Bdh2*, and *Atf5* expression in retinal samples. Since individual rats exhibited differences in expression levels of genes, data represent the mean ± SD obtained for measurements from triplicate wells and are representative of three individual rats. RQ*:* relative quantification.

To ensure the correctness of the pathways from Metascape Analysis, we uploaded the five genes (*Hmox1, Mt1, Slc7a11, Bdh2,* and *Atf5*) to DAVID bioinformatics resources for functional annotation clustering. Based on the functions of the genes assigned by DAVID, genes were classified into the following categories: oxidative stress, negative regulation of neuron apoptotic process, and iron ion homeostasis (Figure 7A). These results suggest that NK-4 up-regulates the genes involved in antioxidative stress and anti-apoptosis pathway.

### 2.4. Validation of RNA-seq Data by RT-qPCR

To validate the reliability of RNA-seq analysis, we selected the five DEGs (*Hmox1, Mt1, Scl7a11, Bdh2,* and *Atf5*) for RT-qPCR analysis. The results of three independent experiments showed increases in the expression levels of the five genes in NK-4-injected samples, compared with control samples. (Figure 7B). Since individual rats exhibited differences in expression levels of genes, data represent the mean ± SD obtained for measurements from triplicate wells and are representative of three individual experiments. 

## 3. Discussion

Previous studies have demonstrated that the cyanine dye NK-4 has neuroprotective effects against oxidative damage in vitro and in vivo [35]. The effects of NK-4 treatment for neurodegenerative diseases have never been used in the field of ophthalmology. We, therefore, injected NK-4 in RCS rats’ eyes to see whether it can protect retinal neurons.

The age of the 3 weeks was chosen for the timing of NK-4 injection to determine whether NK-4 was effective in preventing photoreceptor cells from undergoing apoptosis, because photoreceptors in the RCS rat retina begin to degenerate on postnatal day (P) 22, and the degeneration proceeds rapidly by P32. P22 to P32 is the period with the fastest rate of apoptosis, then the rate of apoptosis slows down [36]. *Mertk* mutation disturbs the microenvironment within the ONL, leading to rapid retinal cell death. The treatment with the NK-4 significantly reduced apoptotic cells at higher concentrations and preserved the thickness of the ONL. Our results demonstrated that intravitreous injection of NK-4 provided protection for the photoreceptor cells at the early onset of dystrophy in RCS rats.

Next, we performed RNA-seq analysis of rat retina to profile the retinal transcriptome following NK-4 injection in RCS rats. We selected five genes (*Hmox1*, *Mt1*, *Slc7a11*, *Bdh2*, *Atf5*) because their upregulation was significant and they were involved in the mechanism of anti-apoptosis. 

Recent studies suggest that regulation of cell metabolism [37], oxidative stress, and metal ion toxicity have been implicated as part of the common pathway in retinal degeneration. Deficiency or excess of metal ions, including Fe, Cu, and Zn, may be caused by genetic mutations and overload of intracellular metal ions, related to the maintenance of retinal metal homeostasis. In RCS rats, a mutation of *Mertk* in RPE may lead to elevated body zinc [38]. The iron imbalance is the result of impaired RPE–photoreceptor interaction, which leads to debris accumulation and subsequent blockage of the iron delivery pathway [39]. The increase in iron in the photoreceptor cells may enhance the vulnerability of cells to oxidative stress [40]. An accumulation of Fe was found in the outer segments and the inner segments of photoreceptors at the age of 35 days [41]. The increase in Fe can cause an overproduction of free radicals and then lead to photoreceptor cell loss.

In this study, *Hmox1* was up-regulated by NK-4. *Hmox1* was reported to promote antioxidant activity and act as a neuroprotective component in neurons [28]. Therefore, *Hmox1* may play a protective role in the early stages of retinal degeneration. 

We also found that metallothionein-1 (*Mt1*) was significantly up-regulated by NK-4, which could protect RPE cells against heme and iron-mediated oxidative damage [29,30].

The cystine/glutamate exchanger *Slc7a11* (system xc^-^) is an important transport system to promote antioxidant signaling in retinal cells [31]. We found that *Slc7a11* was up-regulated by NK-4 in the eyes of RCS rats. The induction of *Slc7a11* expression in the retina by NK-4 may enhance the antioxidant activity to reduce retinal degeneration.

*Bdh2* is expressed in all cell types in the retina to control iron homeostasis [32]. *Bdh2* inhibition was found to result in cellular iron accumulation [33]. In the present study, we found that *Bdh2* was up-regulated in NK-4-injected eyes, indicating that NK-4 may up-regulate the expression of *Bdh2* to protect retina and RPE from damage by reducing excessive iron in the retina of RCS rats. 

Furthermore, we performed GO and KEGG analysis of the DEGs in order to determine the most important transcription factors and pathways influenced by NK-4 injection in the RCS rats. According to the analysis of Metascape software, by uploading the five most interesting genes (*Hmox1*, *Mt1*, *Slc7a11*, *Bdh2*, and *Atf5*), we found that NK-4 injection can up-regulate *Hmox1* expression in pigment metabolic process, transition metal ion homeostasis, and negative regulation of neuron death pathways (Figure 6) and then found that NK-4 injection can up-regulate the inorganic ion homeostasis, transition metal homeostasis, metal ion homeostasis, cation homeostasis pathway, negative regulation of neuron death, neuron death, response to toxic substance, and regulation of neuron death. The upregulation of *Hmox1, Mt1, Slc7a11,* and *Bdh2* genes in these pathways was validated by RT-qPCR and might be related to the antioxidant mechanism. 

Many agents have been tested to slow photoreceptor cell death, such as proinsulin, α-tocopherol, α-lipoic acid, ascorbic acid, a metalloporphyrin superoxide dismutase mimetic, and carnosic acid. α-Tocopherol, α-lipoic acid, and ascorbic acid rescue photoreceptor cells from death due to oxidative damage. Carnosic acid protects retinal neurons through inhibition of endoplasmic reticulum stress and oxidative stress [42,43,44,45]. Clinical trials have shown that long-term supplementation of antioxidants, such as vitamins A and E, β-carotene, docosahexaenoic acid, and zinc, in RP patients improves peripheral visual function [46,47,48]. Anti-apoptotic agents or antioxidants can rescue the photoreceptor cells of RP, providing new insights into the etiology of the loss of photoreceptor cells in the retina, and may open up new therapeutic approaches. Treatment with two molecules, naringenin and quercetin, reduces the levels of ROS in the cellular environment, limiting neurodegeneration, preserving retinal morphology, and improving function [49]. Edaravone inhibited the ONL thinning and reduced the number of TUNEL-positive cells and oxidative stress markers in N-methyl-N-nitrosourea (NMU)-induced retinal photoreceptor degeneration in mice, a model of RP [50,51]. 

Antioxidants can prevent free radical-mediated cell damage and indirectly inhibit cell apoptosis [52]. Oxidative damage can lead to the death of cone cells, and in the more slowly progressive rod degeneration in mouse models of RP, oxidative damage may also lead to the death of rod cells [53]. In RP patients, the death of rod cells reduces oxygen consumption, resulting in higher oxygen levels in the tissues outside the retina. The high level of superoxide free radicals leads to progressive oxidative damage to photoreceptor cells, leading to photoreceptor cell death and loss of function. Preventing oxidative damage may be a widely applicable treatment strategy in RP [54,55,56]. 

Therefore, it is important to develop molecular diagnosis methods for hereditary eye diseases [57] and drugs to simultaneously reduce oxidative stress and apoptosis and then promote the survival function of photoreceptor cells. NK-4 may become a new ophthalmic drug that activates the expression of genes related to antioxidant activity to improve cytoprotective activity and inhibit retinal apoptosis.

This study showed the protective effect of NK-4 on the early stage of the ONL in RCS rats by morphology, RNA sequencing, RT-qPCR. In RNA sequencing, we found that the variation of the expression of selected genes is small. The reason for the low degree of variation is not clear. We selected genes commonly up-regulated (fold change > 1.4) in four independent RNA-seq analyses. As a result, the number of genes selected decreased. In order to see the macro changes on the entire eyeball, the micro changes in the debris clearance, and special mechanisms after NK-4 treatment, we will perform electroretinographic analysis, multicolor immunostaining and, functional experiments in NK-4-treated eyes by long-term treatment in the next step. Further studies are also required to determine whether NK-4 can become a therapeutic strategy that targets a common mechanism of photoreceptor apoptosis among various models of RP and multiple genotypes. 

In summary, our results showed that the injection of NK-4 effectively delayed the apoptosis of photoreceptor cells, postponed the thinning of photoreceptor cells, and exerted a neuroprotective role against retinal apoptosis by promoting anti-apoptotic and antioxidative pathways. These findings provide new insight into molecular mechanisms by which NK-4 exhibits neuroprotective effects in RCS rats and suggest that NK-4 might become a new neuroprotective agent for the prevention and treatment of retinal degeneration.

## 4. Materials and Methods

### 4.1. Chemicals and Preparations

NK-4 was obtained from Hayashibara, Inc. (Okayama, Japan). A stock solution was prepared by mixing NK-4 powder with dimethyl sulfoxide (DMSO) in a flask, heated to dissolve the powder completely, and stored at 4 °C avoiding light. Before use, NK-4 stock solution (5 mg/mL) was diluted with saline to make a series of 10-fold dilutions from 0.0001 to 0.1 mg/mL as working solutions (Figure 1B). Control groups were exposed to an equivalent concentration of DMSO.

### 4.2. Animals 

Animal protocols were approved by the Animal Care and Use Committee of Okayama University, based on the Animal Welfare and Management Act in Japan, and the ARVO Statement for the Use of Animals in Ophthalmic and Vision Research (OKU-2016267 approved on 29 June 2016; OKU-2019196 approved on 1 April 2019). Twenty-three male pink-eyed RCS (Jcl-rdy/rdy, p-) rats were provided by CLEA Japan, Inc. (Tokyo, Japan). The rats at the age of 3 weeks were anesthetized by intraperitoneal injection of ketamine (87 mg/kg body weight) and xylazine (13 mg/kg). Under a dissecting microscope, each rat received an intravitreal injection with a 30-gauge-needle-attached Hamilton syringe (Hamilton Company, Reno, NV, USA) of 5 μL NK-4 working solution in the left eye and vehicle control in the right eye (Figure 1C). Injections were performed on the RCS rats at the ages of 3 weeks and 4 weeks. The needle was inserted perpendicularly at the pars plana, which was about 1.5 mm from the corneal limbus region of the eyes. The rats were housed under a 12 h light/dark cycle for 14 days and then sacrificed at the age of 5 weeks. The eyes were enucleated, immersion-fixed in 4% paraformaldehyde for 3 h, cut into halves along the midperiphery of the eyeballs, immersed in 10% sucrose in 0.1 M phosphate buffer (pH 7.4) for 3 h, and then embedded in OCT compound (Tissue-Tek; Sakura Finetek USA, Torrance, CA, USA). The frozen sections (11 µm thickness) were cut parallel to the vertical meridian of the eye at the optic nerve head with a cryostat. 

### 4.3. TUNEL Staining

Apoptotic cells were detected by terminal deoxynucleotidyl transferase-mediated fluorescein-conjugated dUTP nick-end-labeling (TUNEL) assay (In Situ Cell Death Detection Kit, Fluorescein, Roche Diagnostics, Basel, Switzerland). The study areas of TUNEL staining were located in the sites of a, b, c, d, e and f (“a” and “f”, beginning at 586 μm superiorly and inferiorly from the optic nerve head, respectively; sites “b” and “e”, beginning at 373 μm superiorly and inferiorly from the optic nerve head, respectively; and sites “c” and “d”, beginning at 160 μm superiorly and inferiorly from the optic nerve head, respectively) (Figure 1D). Images (an entire frame (220 × 165 μm)) were collected by the Olympus FSX100 microscope with a 40× objective lens (NA 0.95) and were analyzed by Olympus FSX-BSW software. Three images were obtained for each group. Histological evaluation of the ONL characteristics, such as cell count and ONL thickness, was performed as previously described [58]. TUNEL-positive cells in the ONL for each site (a, b, c, d, e, and f in Figure 1D) were counted and expressed as TUNEL-positive cell number per 1000 µm^2^. In the corresponding area, ONL thickness was manually measured with the layer thicknesses expressed in µm.

### 4.4. RNA Extraction

Neural retinas (right eye, *n* = 4; left eye, *n* = 4) of four rats were dissected under a stereomicroscope. RPE cells attached to the neural retina were carefully removed as much as possible by fine tweezers. The dissected retinas were stored in an RNAlater RNA Stabilization Reagent (Qiagen, Hilden, Germany). Total RNA was extracted from the retina by using the QIAshredder columns and RNeasy Mini Kit (Qiagen). During the extraction, RNA was treated with DNase I set (Qiagen). The quality of RNA was measured by Bioanalyzer 2000.

### 4.5. RNA Sequencing

RNA samples were submitted to the Macrogen Japan and Riken Genesis for Bioanalyzer quality control analysis (QC), Illumina next-generation sequencing (NGS), and differentially expressed gene (DEG) analysis. Qualified samples with RNA integrity number (RIN) > 9 were included for library construction. The sequencing library was prepared with TruSeq Stranded mRNA Library Prep Kit (Macrogen Japan, Kyoto, Japan and Riken Genesis, Tokyo, Japan). Transcriptome sequencing was performed (100 bp paired-end sequencing) on the Novaseq 6000 System (Illumina) and HiSeq 2500 (Illumina) platforms. Cutadapt [59] (version 2.4) removed adapter sequences and low-quality bases from paired reads. The reads were mapped to the rat reference genome (UCSU rn4) by Hisat2 [60] (version 2.1.0), and then transcript assembly was performed by Cufflinks [61] (v2.1.1) using a previously defined rat gene annotation [62]. Differential expression of genes was determined using Cuffdiff in the Cufflinks package. DEGs were explained by an expression change ≥ 1.4-fold and assembled data of four sets (right eye, *n* = 4; left eye, *n* = 4) with a *p* value < 0.05.

### 4.6. Bioinformatics Analysis

Pathway and functional analyses were performed by using the Metascape® (https://metascape.org/, accessed on 30 March 2020). The Database of Essential Genes datasets were uploaded to Metascape, and the networks of the genes were generated based on their connectivity. An upstream regulator analysis was performed to compare DEGs in the datasets to those known to be regulated by a given upstream regulator. Gene Ontology (GO) enrichment and Kyoto Encyclopedia of Genes and Genomes (KEGG) pathway analyses were performed using Database for Annotation, Visualization and Integrated Discovery (DAVID) v6.8 bioinformatics tools (https://david.ncifcrf.gov, accessed on 1 April 2020).

### 4.7. Real-Time Quantitative PCR (RT-qPCR)

cDNA synthesis was performed using a QuantiTect Reverse Transcription Kit (Qiagen) following standard protocols. RT-qPCR was conducted on a 7500 Fast Real-Time PCR System (Applied Biosystems) using SYBR-Green PCR Master Mix. The 2(-Delta Delta C(T)) method was used to analyze the relative changes in the transcript levels of *Hmox1, Mt1, Slc7a11, Bdh2,* and *Atf5* genes. *Beta-actin* genes were used as internal controls for normalizing expression levels of *Hmox1, Mt1,* and *Slc7a11,* and *Tfrc* genes were used as internal controls for normalizing expression levels of *Bdh2 and Atf5* genes. The primers were designed using primer3 plus (http://www.bioinformatics.nl/cgi-bin/primer3plus/primer3plus.cgi, accessed on 2 April 2020) and are listed in Table 2. Measurements were taken in triplicates. 

### 4.8. Statistical Analysis

All values are expressed as mean ± standard deviation (SD). One-way ANOVA with Fisher’s least significant difference (LSD) post hoc test and significant difference in Student’s t-test was used to analyze the differences in multiple comparisons. *p* < 0.05 was considered to be statistically significant. 

## Figures and Tables

**Table 1:** 5 up-regulated genes in NK-4 treated retinas.

Gene Name	Description	Refseq_ID	Locus	FPKM		LogFC	*p* Value
				Control	NK-4		
*Hmox1*	Heme Oxygenase 1	NM_012580	chr19:13963122-13969964	4.36	7.60	0.802	5.75 × 10^−3^
*Mt1*	Metallothionein 1	NM_138826	chr19:11261630-11262647	58.30	114.76	0.977	1.00 × 10^−4^
*Slc7a11*	Solute carrier family 7 member 11	NM_001107673	chr2:139241141-139317101	12.74	21.53	0.756	5.00 × 10^−5^
*Bdh2*	3-hydroxybutyrate dehydrogenase, type 2	NM_001106473	chr2:232765048-232785694	2.01	3.72	0.890	2.46 × 10^−2^
*Atf5*	Activating transcription factor 5	NM_172336	chr1:95284510-95286786	21.24	37.17	0.807	6.00 × 10^−4^

**Table 2:** List of primers used for RT-PCR Analysis.

Gene	Sequence (5’ -> 3’) Forward Primer	Sequence (5’ -> 3’) Reverse Primer
***Hmox1***	cacgcatatacccgctacct	aaggcggtcttagcctcttc
***Mt1***	acctcctgcaagaagagctg	aaactgggtggaggtgtacg
***Scl7a11***	agcagtcccgatctttgttg	aacagctggcagaggagtgt
***Bdh2***	aaccacagagaacggacctg	gacgatgactttcccttcca
***Atf5***	agagggcagagtcagtggaa	ggaagtgaaatggagggaca

## Data Availability

The datasets presented in this study can be found in online repositories. The raw data obtained in this study are available from DDBJ Read Archive (https://ddbj.nig.ac.jp//DRASearch/, accessed on 16 July 2021) under the accession number DRA011674 for RNA-seq.

## Supplementary Materials

The following are available online at https://www.mdpi.com/article/10.3390/ph14070694/s1, Figure S1: TUNEL staining of retinal sections at the site of “a, b, c, d, e”. See Table S1: RNA-seq raw data. Table S2: Annotation of 5 up-regulated genes. Table S3: Enrichment of 5 up-regulated genes.
