# Supplementary material for: The Effect of Cyanine Dye NK-4 on Photoreceptor Degeneration in a Rat Model of Early-Stage Retinitis Pigmentosa
Source: Pharmaceuticals (Basel). 2021 Jul 19;14(7):694. doi: 10.3390/ph14070694 (PMC8308753; doi:10.3390/ph14070694)
Supplement: Supplementary file 1 [file pharmaceuticals-14-00694-s001.zip › rev2_Supplementary Table S2, S3.pdf]

Supplementary Table S2. Annotation of 5 up-regulated genes

MyList	Gene ID	Type	Tax ID	Homologene Gene ID	Homologene Gene Tax ID	Gene Symbol	Description	Biological Process (GO)	GO:0042440 pigment metabolic process	GO:0055076 transition metal ion homeostas	GO:1901215 negative regulation of neuron
hmox1	24451	symbol	R. norvegicus	24451	R. norvegicus	*Hmox1*	heme oxygenase 1	GO:0006788 heme oxidation;GO:0032764 negative regulation of mast cell cytokine production;GO:0034395 regulation of transcription from RNA polymerase II promoter in response to iron	1	1	1
mt1	24567	symbol	R. norvegicus	24567	R. norvegicus	*Mt1*	metallothionein 1	GO:0071247 cellular response to chromate;GO:0046687 response to chromate;GO:0010273 detoxification of copper ion	0	1	1
bdh2	295458	symbol	R. norvegicus	295458	R. norvegicus	*Bdh2*	3-hydroxybutyrate dehydrogenase 2	GO:0009237 siderophore metabolic process;GO:0019290 siderophore biosynthetic process;GO:0019184 nonribosomal peptide biosynthetic process	1	1	0
slc7a11	310392	symbol	R. norvegicus	310392	R. norvegicus	*Slc7a11*	solute carrier family 7 member 11	GO:1901494 regulation of cysteine metabolic process;GO:0031335 regulation of sulfur amino acid metabolic process;GO:1903786 regulation of glutathione biosynthetic process	1	0	1
atf5	282840	symbol	R. norvegicus	282840	R. norvegicus	*Atf5*	activating transcription factor 5	GO:0021891 olfactory bulb interneuron development;GO:0021924 cell proliferation in external granule layer;GO:0021930 cerebellar granule cell precursor proliferation	0	0	0

Supplementary Table S3. Enrichment of 5 up-regulated genes

Group ID	Category	Term	Description	LogP	Log(q-value)	InTerm_InList	Genes	Symbols
1_Summary	GO Biological Processes	GO:0042440	pigment metabolic process	-6.302359406	-2.034	3/67	24451, 295458, 310392	*Hmox1, Bdh2, Slc7a11*
1_Member	GO Biological Processes	GO:0042440	pigment metabolic process	-6.302359406	-2.034	3/67	24451, 295458, 310392	*Hmox1, Bdh2, Slc7a11*
2_Summary	GO Biological Processes	GO:0055076	transition metal ion homeostasis	-5.42142541	-1.454	3/131	24451, 24567, 295458	*Hmox1, Mt1, Bdh2*
2_Member	GO Biological Processes	GO:0055076	transition metal ion homeostasis	-5.42142541	-1.454	3/131	24451, 24567, 295458	*Hmox1, Mt1, Bdh2*
2_Member	GO Biological Processes	GO:0055065	metal ion homeostasis	-3.236322349	0.000	3/708	24451, 24567, 295458	*Hmox1, Mt1, Bdh2*
2_Member	GO Biological Processes	GO:0055080	cation homeostasis	-3.11430281	0.000	3/779	24451, 24567, 295458	*Hmox1, Mt1, Bdh2*
2_Member	GO Biological Processes	GO:0098771	inorganic ion homeostasis	-3.093200204	0.000	3/792	24451, 24567, 295458	*Hmox1, Mt1, Bdh2*
3_Summary	GO Biological Processes	GO:1901215	negative regulation of neuron death	-4.5230839	-0.732	3/261	24451, 24567, 310392	*Hmox1, Mt1, Slc7a11*
3_Member	GO Biological Processes	GO:1901215	negative regulation of neuron death	-4.5230839	-0.732	3/261	24451, 24567, 310392	*Hmox1, Mt1, Slc7a11*
3_Member	GO Biological Processes	GO:1901214	regulation of neuron death	-3.976679761	-0.310	3/398	24451, 24567, 310392	*Hmox1, Mt1, Slc7a11*
3_Member	GO Biological Processes	GO:0070997	neuron death	-3.832640965	-0.263	3/445	24451, 24567, 310392	*Hmox1, Mt1, Slc7a11*
