# Supplementary material for: The Effect of Cyanine Dye NK-4 on Photoreceptor Degeneration in a Rat Model of Early-Stage Retinitis Pigmentosa
Source: Pharmaceuticals (Basel). 2021 Jul 19;14(7):694. doi: 10.3390/ph14070694 (PMC8308753; doi:10.3390/ph14070694)
Supplement: Supplementary file 1 [file pharmaceuticals-14-00694-s001.zip › rev_NK-4_Supplementary figure.pdf]

TUNEL / DAPI (at the site a)

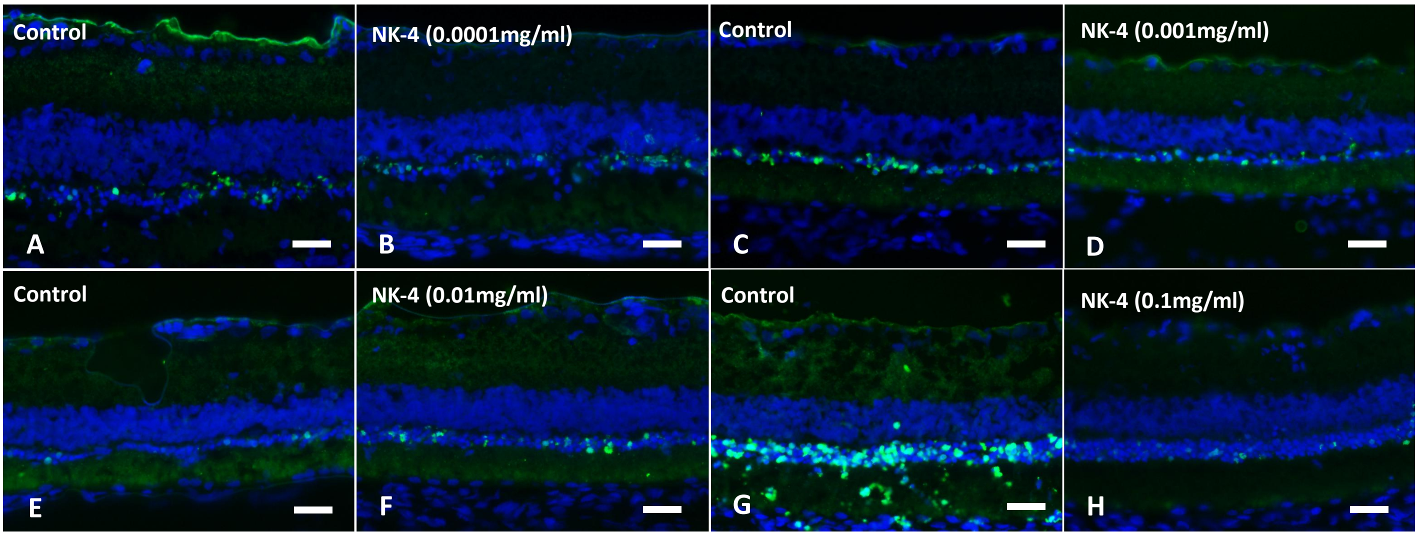

TUNEL / DAPI (at the site b)

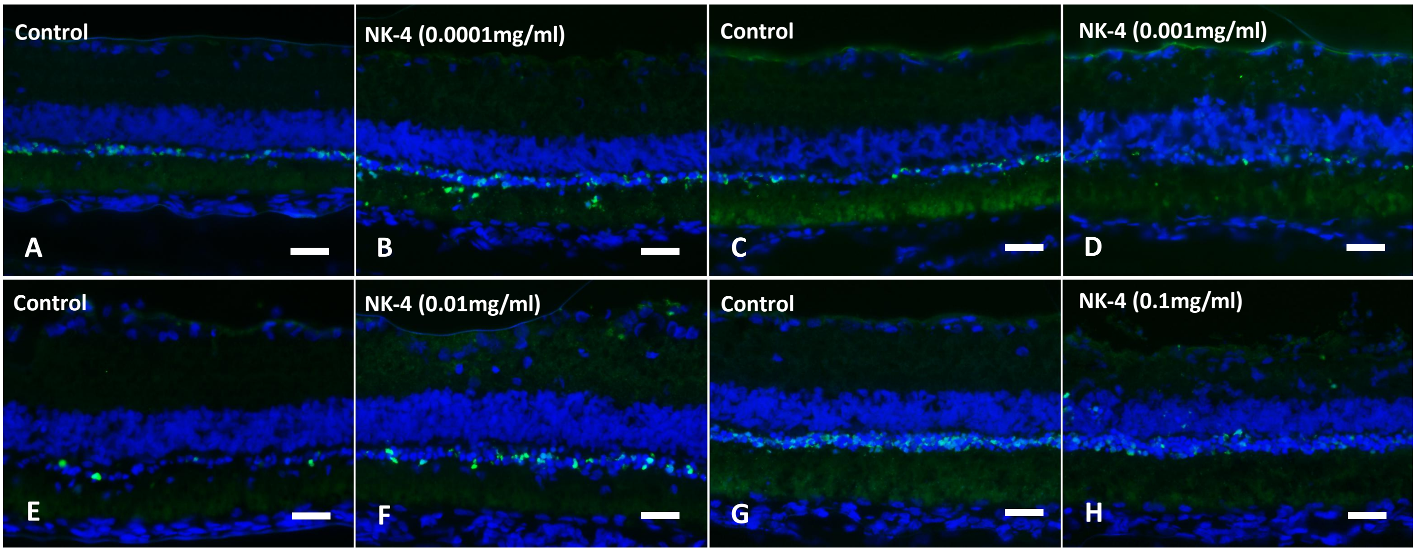

TUNEL / DAPI (at the site c)

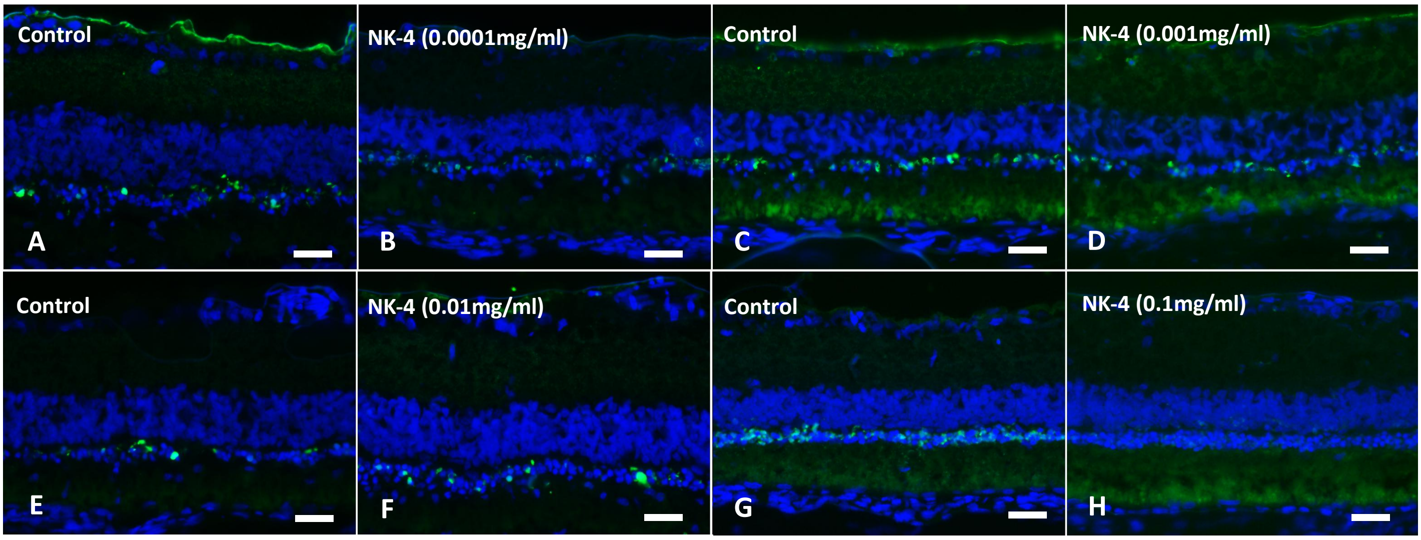

Supplementary Figure S1. TUNEL staining of retinal sections at the site of “a, b, c”. TUNEL assay indicated that TUNEL-positive signals (green) were mainly detected in the ONL and were likely to be fewer in retinas treated with NK-4 (0.0001 mg/ml, 0.001 mg/ml, 0.01 mg/ml, and 0.1 mg/ml) than in those with control vehicle. Cell nuclei were counterstained with DAPI (blue). Pair groups: A vs. B, C vs. D, E vs. F, G vs. H. ONL, outer nuclear layer; INL, inner nuclear layer; OPL, outer plexiform layer. Scale bar = 20 µm.

TUNEL / DAPI (at the site d)

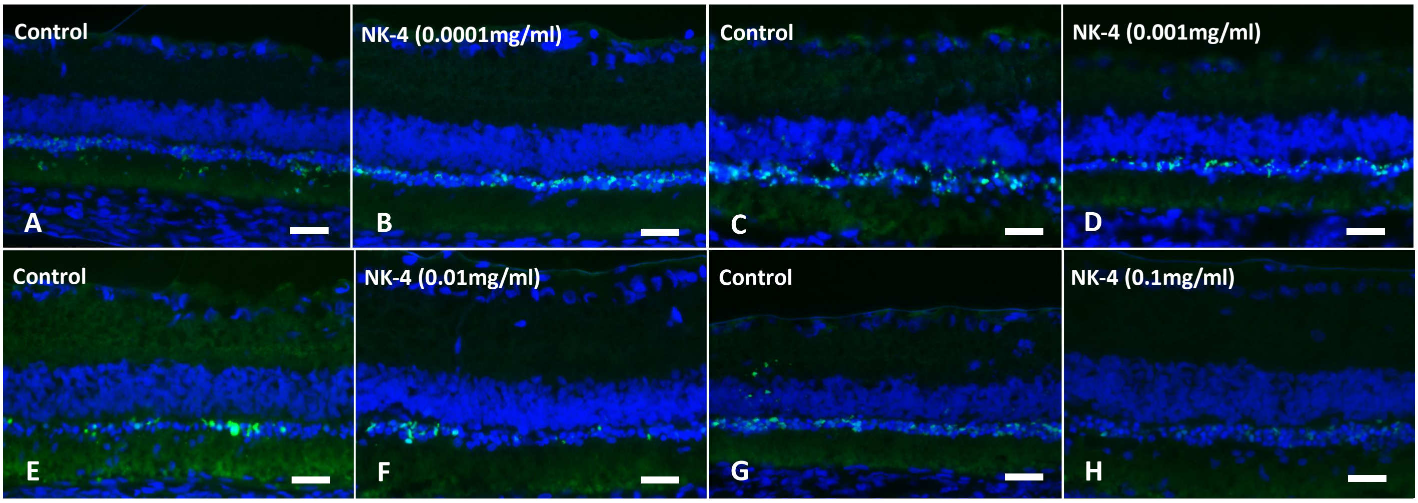

TUNEL / DAPI (at the site e)

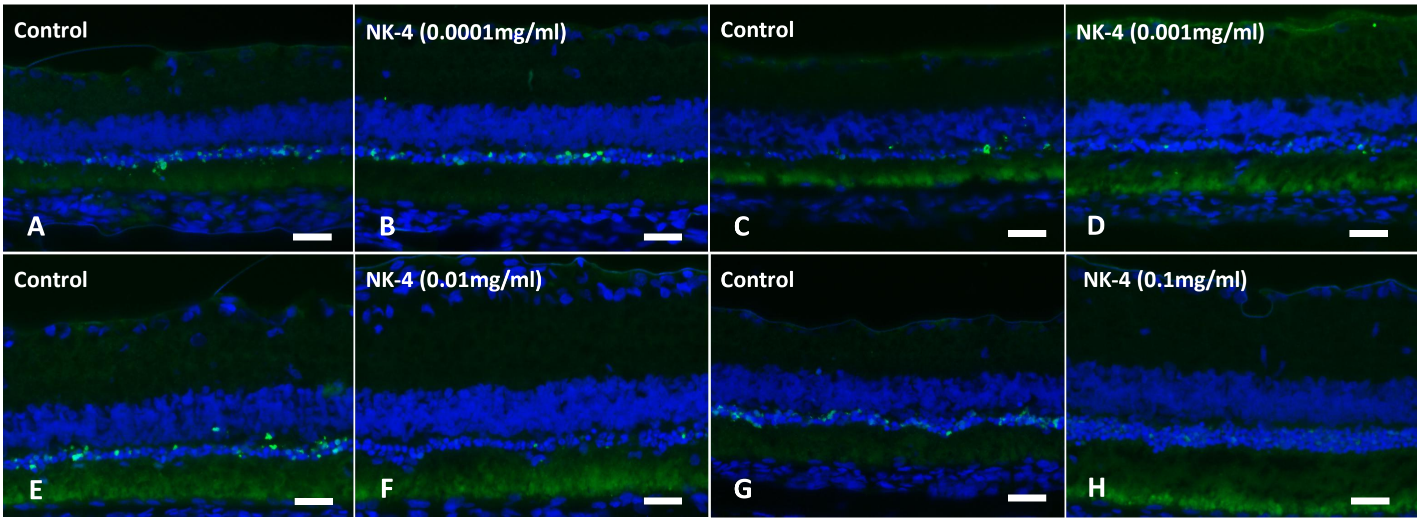

Supplementary Figure S1 (continued). TUNEL staining of retinal sections at the site of “d, e”. TUNEL assay indicated that TUNEL-positive signals (green) were mainly detected in the ONL and were likely to be fewer in retinas treated with NK-4 (0.0001 mg/ml, 0.001 mg/ml, 0.01 mg/ml, and 0.1 mg/ml) than in those with control vehicle. Cell nuclei were counterstained with DAPI (blue). Pair groups: A vs. B, C vs. D, E vs. F, G vs. H. ONL, outer nuclear layer; INL, inner nuclear layer; OPL, outer plexiform layer. Scale bar = 20 µm.
